# L'hidradénite eccrine neutrophilique au cours d'une leucémie aigue myéloïde

**DOI:** 10.11604/pamj.2014.18.72.4570

**Published:** 2014-05-23

**Authors:** Amal Taghy, Badreddine Hassam

**Affiliations:** 1 Service de Dermatologie-Vénérologie, CHU Ibn Sina, Maroc; 2 Faculté de Médecine et de Pharmacie Med V Souissi, Rabat, Maroc

**Keywords:** Leucémie aigue myéloïde, hidradénite, chimiothérapie, Acute myeloid leukemia, hidradenitis, chimiotherapy

## Image en medicine

L'hidradénite eccrine neutrophilique (HEN) appartient au spectre des dermatoses neutrophiliques. Cette pathologie rare touche principalement les adultes traités par chimiothérapie pour une affection onco-hématologique. La physiopathologie reste méconnue à ce jour. Parmi les hypothèses avancées, on suspecte une cytotoxicité des médicaments utilisés au cours des protocoles de chimiothérapie sur les glandes eccrines induisant la production de médiateurs de l'inflammation, ayant une action chimiotactique sur les PNN. Son évolution est finalement bénigne et rapidement résolutive, elle ne doit pas conduire à une modification ou un changement de traitement. Nous rapportons le cas d'un homme de 47 ans, sans antécédents particuliers, traité depuis neuf mois pour leucémie aigue myéloïde et qui avait présenté depuis un mois des lésions palmaires, prurigineuses, érythémato-papuleuses évoluant dans un contexte de fièvre non chiffrée. L'éruption était contemporaine d'une chute importante du taux des polynucléaires neutrophiles (PNN chutent de 8 000 à 500/mm^3^) en cours d'interphase du protocole de chimiothérapie associée à des agents différenciants: acide tout-trans rétinoïque et sels d'arsenic. La biopsie cutanée montrait un infiltrat de polynucléaires neutrophiles à l'intérieur et en périphérie des pelotons des glandes eccrines. L'évolution était marquée par la résolution spontanée des lésions au bout de trois semaines.

**Figure 1:**
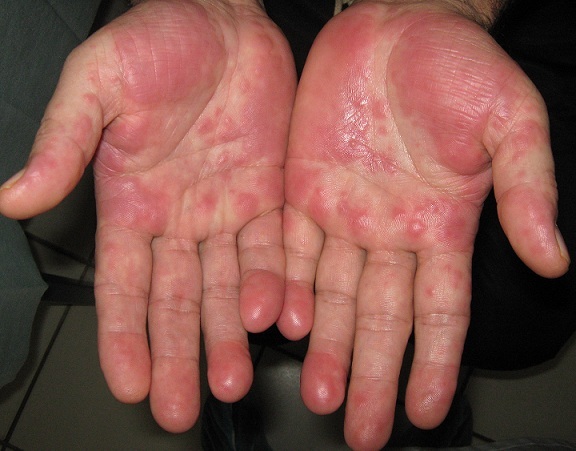
Lésions palmaires érythémato-papuleuses

